# Intravenous ferric carboxymaltose for iron repletion following acute heart failure in patients with and without diabetes: a subgroup analysis of the randomized AFFIRM-AHF trial

**DOI:** 10.1186/s12933-023-01943-z

**Published:** 2023-08-17

**Authors:** Giuseppe Rosano, Piotr Ponikowski, Cristiana Vitale, Stefan D. Anker, Javed Butler, Vincent Fabien, Gerasimos Filippatos, Bridget-Anne Kirwan, Iain C. Macdougall, Marco Metra, Frank Ruschitzka, Vasuki Kumpeson, Udo-Michael Goehring, Peter van der Meer, Ewa A. Jankowska, the AFFIRM-AHF investigators

**Affiliations:** 1 Department of Medical Sciences, Centre for Clinical and Basic Research, IRCCS San Raffaele, Rome, Italy; 2 Institute of Heart Diseases, Wroclaw Medical University, Wroclaw, Poland; 3 Institute of Heart Diseases, University Hospital, Wroclaw, Poland; 4 Department of Cardiology (CVK) of German Heart Center Charité; Institute of Health Center for Regenerative Therapies (BCRT), German Centre for Cardiovascular Research (DZHK) partner site Berlin, Charité Universitätsmedizin, Berlin, Germany; 5 Baylor Scott and White Research Institute, Dallas, TX USA; 6 University of Mississippi, Jackson, MS USA; 7 CSL Vifor, Glattbrugg, Switzerland; 8 National and Kapodistrian University of Athens Medical School, Athens University Hospital Attikon, Athens, Greece; 9 Department of Clinical Research, SOCAR Research SA, Nyon, Switzerland; 10 London School of Hygiene and Tropical Medicine, University College London, London, UK; 11 Department of Renal Medicine, King’s College Hospital, London, UK; 12 Cardiology, ASST Spedali Civili and Department of Medical and Surgical Specialties, Radiological Sciences, and Public Health, University of Brescia, Brescia, Italy; 13 Department of Cardiology, University Heart Center, University Hospital Zurich and University of Zurich, Zurich, Switzerland; 14 Department of Cardiology, University Medical Center Groningen, Groningen, The Netherlands

**Keywords:** Diabetes, Acute heart failure, Iron deficiency, Ferric carboxymaltose, AFFIRM-AHF

## Abstract

**Background:**

In AFFIRM-AHF, treatment of iron deficiency with intravenous ferric carboxymaltose (FCM) reduced the risk of heart failure (HF) hospitalization and improved quality of life (QoL) vs placebo in patients stabilized following an acute HF (AHF) episode, with no effect on cardiovascular (CV) death. Diabetes and iron deficiency frequently accompany AHF. This post hoc analysis explored the effects of diabetes on outcomes in AFFIRM-AHF patients.

**Methods:**

Patients were stratified by diabetes yes/no at baseline. The effects of FCM vs placebo on primary (total HF hospitalizations and CV death) and secondary (total CV hospitalizations and CV death; CV death; total HF hospitalizations; time to first HF hospitalization or CV death; and days lost due to HF hospitalizations or CV death) endpoints at Week 52 and change vs baseline in disease-specific QoL (12-item Kansas City Cardiomyopathy Questionnaire [KCCQ-12]) at Week 24 were assessed by subgroup. For each endpoint, the interaction between diabetes status and treatment outcome was explored.

**Results:**

Of 1108 AFFIRM-AHF patients, 475 (FCM: 231; placebo: 244) had diabetes and 633 (FCM: 327; placebo: 306) did not have diabetes. Patients with diabetes were more commonly male (61.5% vs 50.9%), with a higher frequency of ischemic HF etiology (57.9% vs 39.0%), prior HF history (77.7% vs 66.5%), and comorbidities (including previous myocardial infarction [49.3% vs 32.9%] and chronic kidney disease [51.4% vs 32.4%]) than those without diabetes. The annualized event rate/100 patient-years with FCM vs placebo for the primary endpoint was 66.9 vs 80.9 in patients with diabetes (rate ratio [RR]: 0.83, 95% CI 0.58–1.81) and 51.3 vs 66.9 in patients without diabetes (RR: 0.77, 95% CI 0.55–1.07), with no significant interaction between diabetes status and treatment effect (p_interaction_ = 0.76). Similar findings were observed for secondary outcomes. Change from baseline in KCCQ-12 overall summary score was numerically greater with FCM vs placebo at almost all time points in both subgroups, with no interaction between diabetes and treatment effect at Week 24.

**Conclusions:**

The clinical and QoL benefits observed with intravenous FCM in patients with iron deficiency following stabilization from an AHF episode are independent of diabetes status.

*Trial registration* Clinicaltrials.gov, NCT02937454 (registered 10.18.2016).

**Supplementary Information:**

The online version contains supplementary material available at 10.1186/s12933-023-01943-z.

## Background

Acute heart failure (AHF) remains a leading cause of hospitalization, especially in the elderly and those with a history of heart failure (HF), and is associated with high mortality and rehospitalization rates [1–4]. Diabetes and HF frequently occur together, with diabetes affecting approximately 30–50% of HF patients [5–11], despite a potential underdiagnosis of type 2 diabetes in this population [12]. Patients with diabetes are at a greater risk of developing HF and vice versa [13]. Patients with HF and diabetes have a poorer quality of life (QoL) and higher rates of HF rehospitalization and mortality than patients with HF without diabetes [5, 7–10, 14, 15]. Consequently, the European Society of Cardiology and European Association for the Study of Diabetes recommend studies to better understand the bidirectional relationship between HF and diabetes, and improve HF outcomes in patients with these co-existing conditions [13].

Iron deficiency is common in both acute and chronic HF [16–19] and is associated with increased risk of hospitalization and death, as well as impaired QoL and exercise tolerance [20]. In patients without diabetes, there is some evidence linking iron deficiency with elevated glycated Hb (HbA_1c_) levels [21, 22]. Additionally, diabetes is significantly more prevalent in patients with HF who have iron deficiency compared with those who have normal iron levels [18], and there is some evidence linking a longer diabetes duration with iron deficiency in patients with diabetes and cardiovascular disease [23]. In addition, the impaired renal function that often accompanies diabetes and contributes to the pro-inflammatory disease state, potentially disrupts gastrointestinal absorption and mobilization of iron [24]. The effects of treating iron deficiency in patients with HF and co-existing diabetes are, therefore, of clinical interest.

The AFFIRM-AHF trial (NCT02937454) reported that, in patients stabilized following an AHF episode, treating iron deficiency with intravenous (IV) ferric carboxymaltose (FCM) significantly reduced the risk of HF hospitalizations and improved QoL, without affecting the risk of cardiovascular death, compared with placebo [19, 25]. Here, we report an AFFIRM-AHF post hoc analysis that aimed to explore the effect of diabetes status on treatment outcomes with FCM vs placebo.

## Methods

The design and primary results of the international, multicenter, double-blind, placebo-controlled, phase 4 randomized AFFIRM-AHF trial are already published [19, 26]. The trial was conducted in accordance with the Declaration of Helsinki, the International Conference on Harmonization Good Clinical Practice guidelines, and local and national regulations. The relevant ethical review boards approved the protocol, and all patients provided their written informed consent to participate.

AFFIRM-AHF included patients aged ≥ 18 years who had been hospitalized with signs and symptoms typical of AHF, treated with a minimum of 40 mg IV furosemide (or equivalent IV diuretic), and who had concomitant iron deficiency (defined as serum ferritin < 100 μg/L, or serum ferritin 100–299 μg/L with transferrin saturation [TSAT] < 20%) and a left ventricular ejection fraction < 50%. Patients were randomly assigned (1:1) to receive IV FCM or placebo, with the first dose administered shortly before discharge and the second dose administered at Week 6 (dose based on screening hemoglobin [Hb] and body weight values, as detailed previously [19]). At Weeks 12 and 24, only patients in whom iron deficiency persisted and for whom Hb was 8–15 g/dL were administered study drug. Patients were followed for a further 28 weeks without study drug treatment, up to Week 52. In this post hoc subgroup analysis, patients were stratified according to the investigator-indicated diabetes status (yes/no) in the AFFIRM-AHF electronic clinical report form (eCRF). The use of diabetes medication at baseline in those with diabetes status “no” in the eCRF was then examined to assess the need for reclassification into the diabetes subgroup.

The primary endpoint was a composite of total HF hospitalizations and CV death up to 52 weeks of follow-up. Secondary clinical endpoints (total CV hospitalizations and CV death; CV death; total HF hospitalizations; time to first HF hospitalization or CV death; and days lost due to HF hospitalizations or CV death) were also evaluated up to 52 weeks. Other endpoints included changes in disease-specific QoL (assessed using the self-administered 12-item Kansas City Cardiomyopathy Questionnaire [KCCQ-12] overall summary score [OSS] and clinical summary score [CSS]) from baseline to Weeks 2, 4, 6, 12, 24, 36, and 52, and laboratory values (serum ferritin, Hb, and TSAT) from baseline to Weeks 6, 12, 24, and 52. Safety endpoints included a summary of adverse events (AEs).

All analyses were based on data for AFFIRM-AHF patients with known diabetes status at baseline, with the safety analysis set (SAS) used for safety and laboratory endpoint analyses and the modified intention-to-treat (mITT) population used for all other endpoint analyses. Given the limited number of patients in the subgroups stratified by diabetes status, baseline characteristics were descriptively summarized as mean (standard deviation [SD]) for continuous variables and n (%) for discrete variables, and statistical significance was not assessed. Chronic kidney disease was determined by investigator-indicated status (yes/no) in the AFFIRM-AHF eCRF. Primary and secondary outcomes with FCM vs placebo within each subgroup were analyzed using a negative binomial model for recurrent endpoints (presented as event rate ratios [RRs] and 95% confidence intervals [CIs]) and a Cox regression model for time to first event endpoints (presented as hazard ratios and 95% CIs). P-values for treatment effect within the subgroups by diabetes status are nominal and descriptive only. Interaction p-values (p_interaction_) for the effect of diabetes status on treatment outcomes were generated. As previously described, a prespecified pre-COVID-19 sensitivity analysis, which censored patients in each country at the date when its first COVID-19 patient was reported, was also carried out to account for the impact of COVID-19 on primary and secondary outcomes [19]. To assess the impact of diabetes in patients that did not receive FCM, primary and secondary outcomes were also compared in the placebo arms of each diabetes subgroup.

In each diabetes subgroup, mean (standard error) changes from baseline in KCCQ-12 OSS and CSS and in laboratory values (serum ferritin, Hb, and TSAT) with FCM vs placebo were compared at each time point using repeated measures ANOVA. P-values for treatment effect within the subgroups by diabetes status are nominal and descriptive only. Interaction p-values evaluating the interaction between diabetes status and the effect of FCM vs placebo on KCCQ-12 OSS and CSS at Week 24 (end of treatment period) were generated. AEs were descriptively summarized in each subgroup and treatment arm as number of subjects with events (%) and number of events. Analyses were not adjusted for multiplicity. For all analyses, SAS version 9.4 (SAS Institute, Inc., Cary, NC, USA; 2000–2004) was used, with p-values < 0.05 considered statistically significant.

## Results

### Baseline characteristics, by diabetes status

From the 1108 patients in the main AFFIRM-AHF mITT analysis with documented diabetes status in the eCRF, 470 (FCM: 227; placebo: 243) patients were recorded as having diabetes (yes) by the project investigator. Of these patients, two in the placebo arm had type 1 and the remainder had type 2 diabetes. An additional five patients (four in the FCM and one in the placebo arm) with a diabetes status of “no” recorded in the eCRF were receiving diabetes medications at baseline for other documented glycemic irregularities (hyperglycemia in one patient; irregular sugar curve in one patient; glucose intolerance in three patients); these five patients were reclassified into the diabetes subgroup for the purposes of these analyses. As such, the mITT diabetes subgroup consisted of 475 (FCM: 231; placebo: 244) patients and the mITT no diabetes subgroup consisted of 633 (FCM: 327; placebo: 306) patients.

Baseline characteristics by diabetes status are shown in Table 1. Patients with diabetes were more commonly male (61.5% vs 50.9%), with a numerically higher frequency of other comorbidities such as previous myocardial infarction (49.3% vs 32.9%), stroke (12.6% vs 9.3%), or chronic kidney disease (51.4% vs 32.4%) than those without diabetes. Patients with diabetes also had a numerically higher frequency of ischemic HF etiology (57.9% vs 39.0%), prior HF history (77.7% vs 66.5%), and hospitalization for HF in the previous 12 months (43.6% vs 34.2%) than those without diabetes. Amongst patients with diabetes, the most common diabetes medications were insulin and insulin analogs (53.7%), biguanides (40.8%), and sulfonylureas (22.3%); use of sodium–glucose co-transporter inhibitors (3.2%) and glucagon-like peptide-1 receptor agonists (1.1%) was less common. Baseline HbA_1c_ data were available for only 15 subjects and are therefore not presented here.

**Table 1:** Baseline demographics and clinical characteristics by baseline diabetes status (mITT)

Baseline characteristics	Diabetes (N = 475)	No diabetes (N = 633)
Age, years	69.9 (9.7)	71.9 (11.8)
Sex, n (%)		
Male	292 (61.5)	322 (50.9)
Female	183 (38.5)	311 (49.1)
Race, n (%)		
White	437 (92.0)	614 (97.0)
Asian	30 (6.3)	18 (2.8)
Other	8 (1.7)	1 (0.2)
Comorbidities, n (%)		
Previous myocardial infarction	234 (49.3)	208 (32.9)
Previous stroke	60 (12.6)	59 (9.3)
Previous coronary revascularization	101 (21.3)	69 (10.9)
Hypertension	429 (90.3)	510 (80.6)
Atrial fibrillation	253 (53.3)	366 (57.8)
Diabetes	475 (100.0)	0 (0)
Dyslipidemia	314 (66.1)	278 (43.9)
Chronic kidney disease	244 (51.4)	205 (32.4)
Smoking	216 (45.5)	203 (32.1)
Systolic blood pressure, mmHg (SD)	121 (16)	119 (15)
Diastolic blood pressure, mmHg (SD)	72 (10)	73 (10)
Heart rate, beats per minute (SD)	73.7 (12.8)	74.8 (13.1)
NYHA classification, n (%)		
≤ Class II	212 (44.6)	305 (48.2)
≥ Class III	262 (55.2)	325 (51.3)
Left ventricular ejection fraction, % (SD)	32 (10)	33 (9)
Left ventricular ejection fraction, n (%)		
< 25%	111 (23.4)	115 (18.2)
≥ 25% to < 40%	213 (44.8)	318 (50.2)
≥ 40% to < 50%	150 (31.6)	200 (31.6)
Ischemic HF, n (%)	275 (57.9)	247 (39.0)
Device therapy, n (%)		
Implantable cardioverter-defibrillator	70 (14.7)	61 (9.6)
Cardiac resynchronization therapy	30 (6.3)	33 (5.2)
Heart failure history, n (%)		
Newly diagnosed at index hospitalization	106 (22.3)	212 (33.5)
Documented history of HF	369 (77.7)	421 (66.5)
Hospitalization for HF in previous 12 months	161 (43.6)	144 (34.2)
Pharmacotherapy, n (%)		
ACEi	232 (48.8)	344 (54.3)
ARB	85 (17.9)	112 (17.7)
ARNI	36 (7.6)	35 (5.5)
Aldosterone antagonist	299 (62.9)	429 (67.8)
Beta blocker	405 (85.3)	509 (80.4)
Digitalis glycosides	80 (16.8)	104 (16.4)
Loop diuretic	419 (88.2)	529 (83.6)
Laboratory test results		
NT-pro-BNP, pg/mL (median [upper and lower quartiles])	4675 (2839; 8506)	4743 (2754; 8338)
BNP, pg/mL (median [upper and lower quartiles])	1068 (810; 1667)	1195 (796; 1821)
Hb, g/dL	12.0 (1.6)	12.3 (1.6)
Hb category, n (%)		
< 10 g/dL	56 (11.8)	58 (9.2)
≥ 10 to < 14 g/dL	366 (77.1)	466 (73.6)
≥ 14 g/dL	53 (11.2)	108 (17.1)
Serum ferritin, ng/mL	90.7 (67.0)	82.7 (64.2)
Serum ferritin < 100 ng/mL, n, (%)	323 (68.0)	465 (73.5)
TSAT, %	13.8 (6.2)	15.4 (8.9)
TSAT < 20%, n (%)	419 (88.2)	507 (80.1)
eGFR, mL/min per 1.73 m^2^	53.6 (22.9)	56.9 (21.5)
Phosphorous, mg/dL	3.8 (0.9)	3.7 (0.8)
Diabetes medication		
Insulins and analogs	255 (53.7)	0 (0)
Biguanides	194 (40.8)	0 (0)
Sulfonylurea	106 (22.3)	0 (0)
Dipeptidyl peptidase 4 inhibitor	31 (6.5)	0 (0)
Combinations of oral BG-lowering drugs	22 (4.6)	0 (0)
Sodium–glucose co-transporter 2 inhibitor	15 (3.2)	0 (0)
Alpha glucosidase inhibitors	8 (1.7)	0 (0)
Glucagon-like peptide-1 analog	5 (1.1)	0 (0)
Thiazolidinediones	1 (0.2)	0 (0)
Number of diabetes medications		
0	89 (18.7)	633 (100)
1	184 (38.7)	0 (0)
2	140 (29.5)	0 (0)
≥ 3	62 (13.1)	0 (0)

Chronic kidney disease was determined by investigator-indicated status (yes/no) in the AFFIRM-AHF eCRF. Baseline medication was defined as any medication that was current on the initial dosing of study drug. Data are mean (SD) unless otherwise specified

*ACEi* angiotensin-converting enzyme inhibitor, *ARB* angiotensin receptor blocker, *ARNI* angiotensin receptor-neprilysin inhibitor, *BG* blood glucose, *BNP* brain natriuretic peptide, *eGFR* estimated glomerular filtration rate, *Hb* hemoglobin, *HF* heart failure, *mITT* modified intention-to-treat, *NT-pro-BNP* N-terminal-pro brain natriuretic peptide, *NYHA* New York Heart Association, *SD* standard deviation, *TSAT* transferrin saturation

### Treatment exposure by diabetes status

**Fig. 1:**
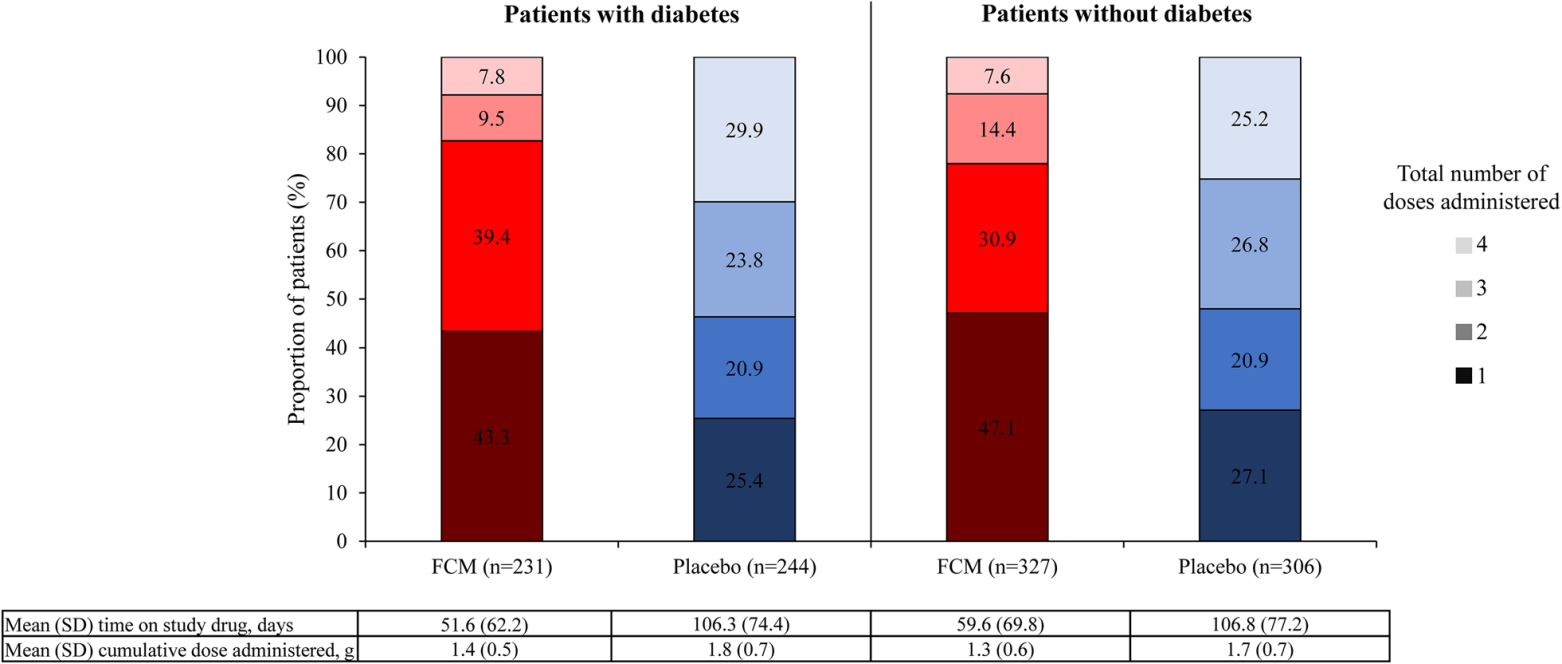
Treatment exposure by diabetes status. Time on study drug calculated from the date of the first study drug administration to the date of the last study drug injection plus 1 day. *FCM* ferric carboxymaltose; *SD* standard deviation

Figure 1 shows the study drug exposure by diabetes subgroup and treatment arm. Irrespective of diabetes status, the proportion of patients with persisting iron deficiency at Week 12 and/or 24 who were therefore eligible for a third and/or fourth dose of study drug according to the study protocol was higher in the placebo arm compared with the FCM arm (53.7% vs 17.3% in the diabetes subgroup; 52.0% vs 22.0% in the no diabetes subgroup). The mean (SD) cumulative dose of study drug administered was also numerically higher in the placebo arm compared with the FCM arm of each subgroup (1.8 g [0.7] vs 1.4 g [0.5], respectively, in patients with diabetes; 1.7 g [0.7] vs 1.3 g [0.6], respectively, in patients without diabetes). The mean (SD) number of days on study drug (calculated from the date of the first study drug administration to the date of the last study drug injection plus 1 day) in the placebo and FCM arms were 106.3 (74.4) and 51.6 (62.2) days, respectively, in patients with diabetes and 106.8 (77.2) and 59.6 (69.8) days, respectively, in those without diabetes.

### Primary and secondary outcomes, by diabetes status

In the placebo arm, the adjusted, annualized event rate for the primary outcome was nominally higher in patients with diabetes than in patients without diabetes (66.9 vs 54.3 per 100 patient-years; RR: 1.23, 95% CI 0.88–1.72); similar results were observed for secondary outcomes (Additional file 1: Fig S1).

**Fig. 2:**
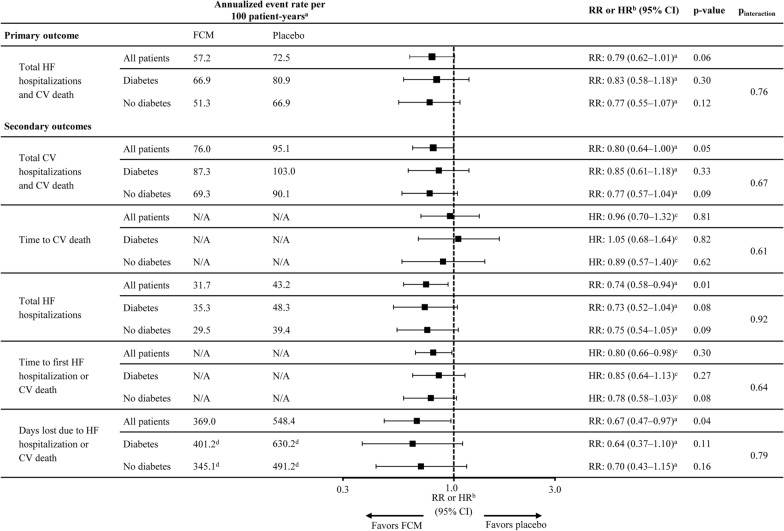
Primary and secondary outcomes with FCM vs placebo in patients with and without diabetes. mITT population analyzed. All models adjusted for covariates: sex, age, HF etiology, HF duration, country, and diabetes status at baseline, and diabetes at baseline × treatment. N = 1108 for all patients. Respective n-values in patients with and without diabetes at baseline were 231 and 327 for FCM and 244 and 306 for placebo. ^a^Annualized event rate per 100 patient-years and annualized event RR were both analyzed using a negative binomial model. ^b^FCM vs placebo. ^c^HR for treatment difference analyzed using Cox regression model. ^d^Event refers to days off work. *CI* confidence interval, *CV* cardiovascular, *FCM* ferric carboxymaltose, *HF* heart failure, *HR* hazard ratio, *mITT* modified intention-to-treat, *RR* rate ratio

Regarding treatment effect, reductions in annualized event rates with FCM vs placebo were observed in patients both with and without diabetes (Fig. 2). The annualized event rate per 100 patient-years for the primary outcome in the FCM vs placebo arm was 66.9 vs 80.9 in patients with diabetes (RR: 0.83; 95% CI 0.58‒1.18) and 51.3 vs 66.9 in those without diabetes (RR: 0.77; 95% CI 0.55‒1.07). There was no significant interaction between diabetes status at baseline and treatment effect for the primary outcome (p_interaction_ = 0.76). Similar findings were observed for secondary outcomes (Fig. 2) and for the pre-COVID-19 sensitivity analysis (Additional file 1: Fig S2).

### Disease-specific QoL, by diabetes status

**Fig. 3:**
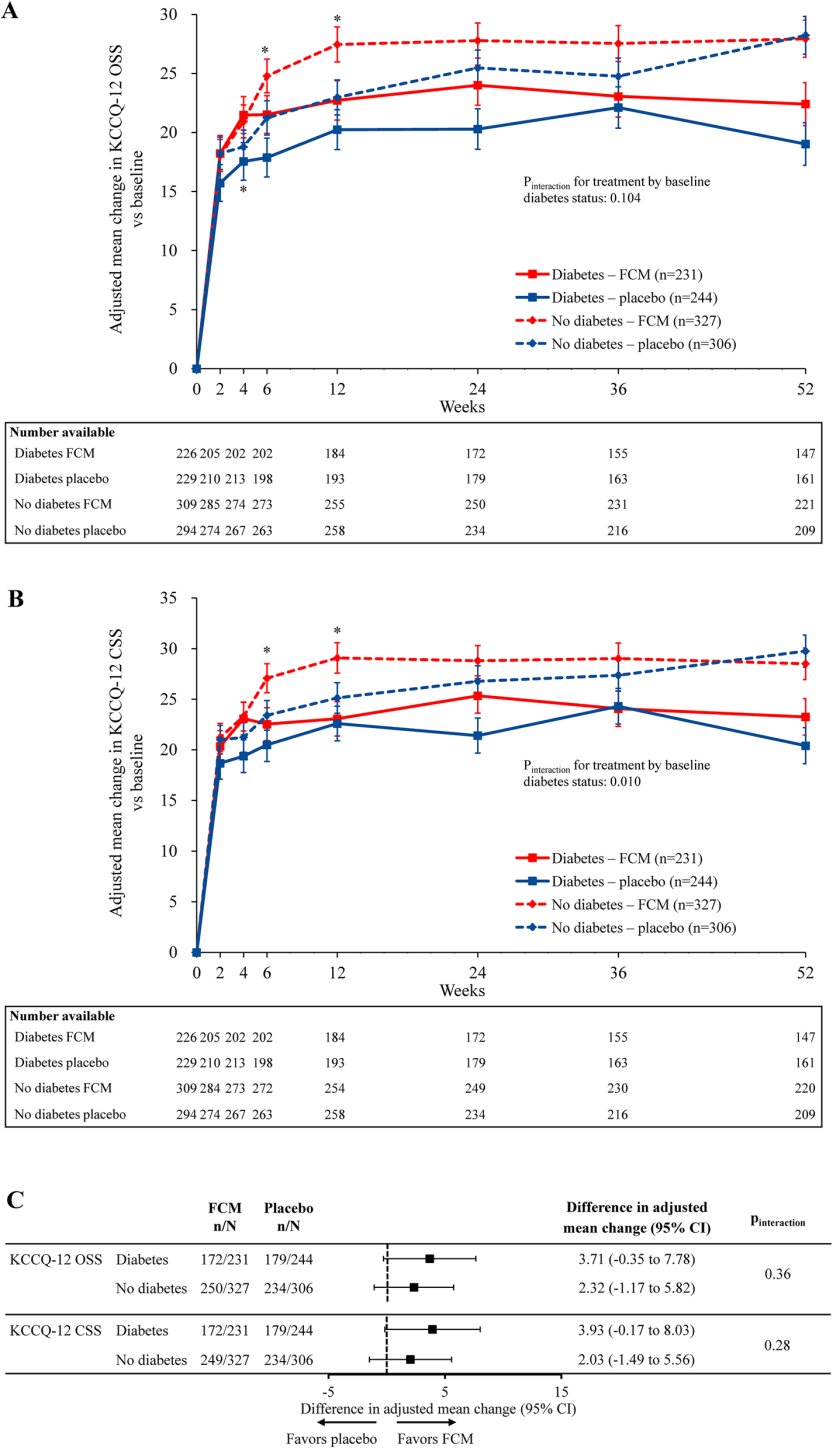
KCCQ-12 OSS and CSS with FCM vs placebo in patients with and without diabetes. KCCQ-12 OSS (**A**) and CSS (**B**) with FCM vs placebo in patients with and without diabetes, and (**C**) interaction of diabetes status with FCM treatment effect at Week 24. mITT population analyzed. *p < 0.05 for FCM vs placebo no diabetes subgroup only (no significant changes were observed in the diabetes subgroup). Error bars are standard error. Estimates are based on a mixed-effect model of repeated measures using an unstructured covariance matrix: change score = baseline score + treatment + visit + treatment × visit + subgroup + subgroup × visit + subgroup × treatment + subgroup × treatment × visit + baseline covariates. *CI* confidence interval, *CSS* clinical summary score, *FCM* ferric carboxymaltose, *KCCQ-12* Kansas City Cardiomyopathy Questionnaire, *mITT* modified intention-to-treat, *OSS* overall summary score

Mean (SD) baseline KCCQ-12 OSS scores were similar across treatment arms and diabetes status subgroups (diabetes: 38.3 [20.5] FCM, 37.5 [19.3] placebo; no diabetes: 37.9 [19.4] FCM, 36.9 [18.6] placebo), as were baseline KCCQ-12 CSS scores (diabetes: 40.8 [21.7] FCM, 40.6 [20.2] placebo; no diabetes: 41.0 [20.0] FCM, 39.7 [20.0] placebo). Figure 3 shows the adjusted mean change from baseline in KCCQ-12 OSS (Fig. 3A) and CSS (Fig. 3B) over time by diabetes status and treatment group, as well as the interaction between these variables and KCCQ-12 score outcomes at Week 24 (Fig. 3C). In patients with and without diabetes, visually greater improvements in KCCQ-12 OSS and CSS were observed with FCM vs placebo at the majority of time points. There were no significant interactions between diabetes status at baseline and the effect of FCM vs placebo on KCCQ-12 OSS (p_interaction_ = 0.36) or KCCQ-12 CSS (p_interaction_ = 0.28) at Week 24.

### Iron parameters over time, by diabetes status

**Fig. 4:**
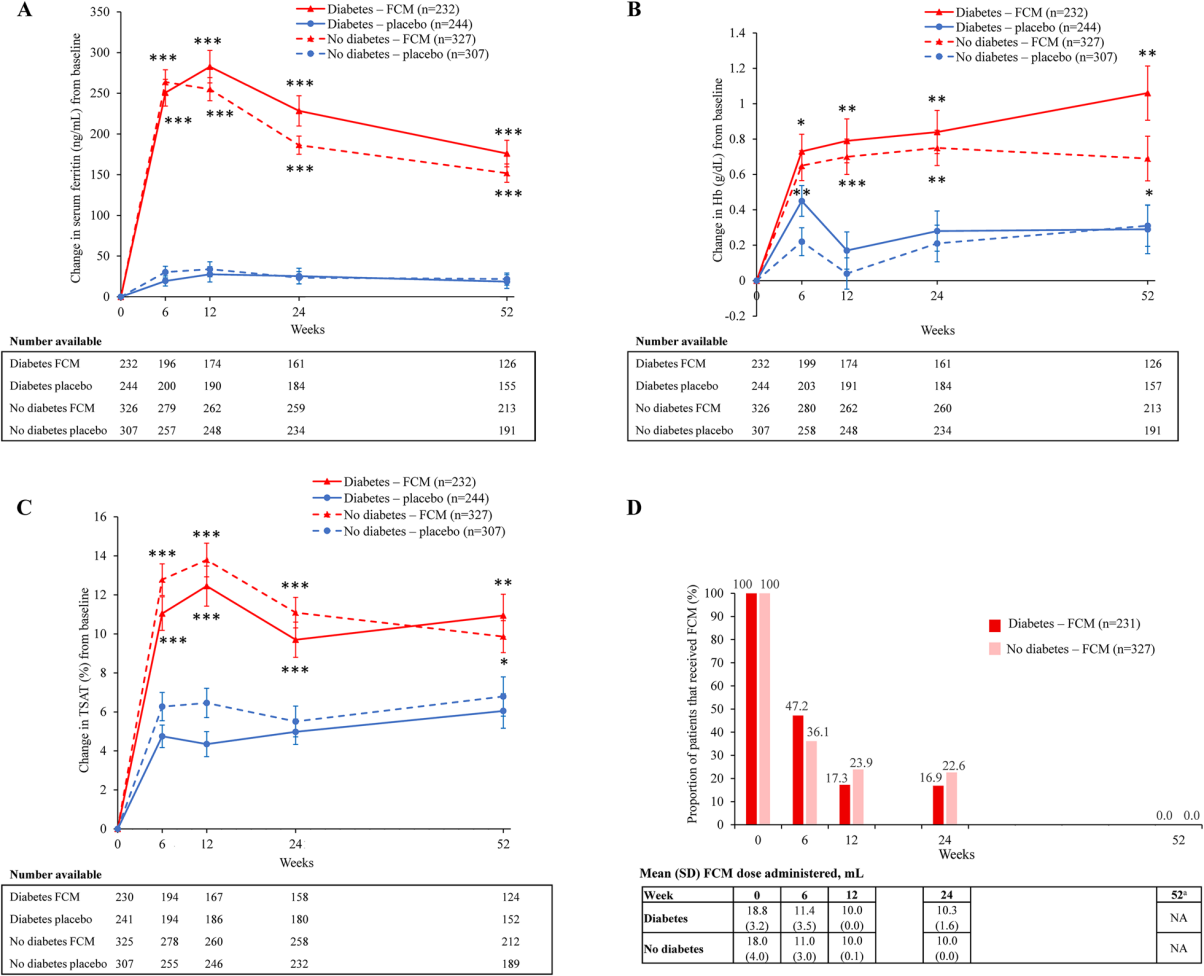
Iron parameters with FCM vs placebo in patients with and without diabetes. Absolute change from baseline in (**A**) serum ferritin, (**B**) hemoglobin, and (**C**) TSAT over time with FCM vs placebo (SAS population), and (**D**) FCM dosing at each time point in patients with and without diabetes (mITT population). Error bars are standard error of the mean. *p < 0.05, **p < 0.01, and ***p < 0.0001 for FCM vs placebo. ^a^No study drug was administered after Week 24, as per the protocol. *FCM* ferric carboxymaltose, *Hb* hemoglobin, *mITT* modified intention-to-treat, *NA* not applicable, *SAS* safety analysis set, *SD* standard deviation, *TSAT* transferrin saturation

Figure 4 and Additional file 1: Fig S3 summarize the changes in iron parameters in patients with and without diabetes receiving FCM or placebo. Serum ferritin, Hb, and TSAT levels increased to a significantly greater magnitude with FCM compared with placebo in patients with and without diabetes at all time points.

### First-time initiation of diabetes medications during the study

The proportions of patients with first-time initiation of a therapy within a particular diabetes medication class during the trial were similar between FCM and placebo arms (Additional file 1: Table S1).

### Summary of adverse events

Data on AEs and treatment-emergent AEs (TEAEs) in the SAS can be found in Table 2. In general, AEs were reported for a higher proportion of patients in the diabetes subgroup (74.2% [1338 events in 353/476 patients]) compared with the no diabetes subgroup (62.3% [1332 events in 395/634 patients]); observations were similar for serious TEAEs (55.9% [620 events in 266/476 patients] and 42.0% [559 events in 266/634 patients], respectively). The proportion of patients with ≥ 1 TEAE was similar with FCM vs placebo in each subgroup. No fatal TEAEs related to the study drug were observed in either subgroup.

**Table 2:** Summary of adverse events by baseline diabetes status (SAS)

Adverse events	Diabetes (N = 476)				No diabetes (N = 634)			
	FCM (n = 232)		Placebo (n = 244)		FCM (n = 327)		Placebo (n = 307)	
	Subjects, n (%)	Events, n	Subjects, n (%)	Events, n	Subjects, n (%)	Events, n	Subjects, n (%)	Events, n
All adverse events	167 (72.0)	619	186 (76.2)	719	207 (63.3)	680	188 (61.2)	652
All TEAEs	163 (70.3)	601	181 (74.2)	693	194 (59.3)	645	179 (58.3)	621
Related to study drug	9 (3.9)	12	0	0	3 (0.9)	3	2 (0.7)	2
Leading to treatment discontinuation	35 (15.1)	39	46 (18.9)	52	26 (8.0)	32	33 (10.7)	36
Leading to hospitalization	106 (45.7)	237	132 (54.1)	312	120 (36.7)	246	125 (40.7)	249
Leading to study discontinuation	52 (22.4)	61	47 (19.3)	59	46 (14.1)	56	49 (16.0)	64
Serious TEAEs	120 (51.7)	272	146 (59.8)	348	130 (39.8)	275	136 (44.3)	284
Related to study drug	1 (0.4)	3	0	0	0	0	2 (0.7)	2
Fatal TEAEs	52 (22.4)	61	48 (19.7)	60	47 (14.4)	57	48 (15.6)	63
Related to study drug	0	0	0	0	0	0	0	0

Related TEAEs are defined as TEAEs that are considered at least possibly related to the study product. Percentage of subjects is computed with respect to the number of subjects by treatment group in the SAS

*FCM*, ferric carboxymaltose, *SAS* safety analysis set, *TEAE* treatment-emergent adverse event

## Discussion

This post hoc analysis of the AFFIRM-AHF trial showed that the benefits of IV FCM vs placebo for reducing cardiovascular outcomes (including HF hospitalizations) and improving QoL in patients with iron deficiency following stabilization of an AHF episode were irrespective of diabetes status.

Multiple studies have reported greater morbidity and mortality in patients with HF and diabetes than in patients with HF and no diabetes [5, 7–10, 14, 15]. The data from this analysis support these prior findings: in general, patients with diabetes tended to have more severe and more chronic HF with a higher prevalence of other comorbidities compared with patients without diabetes. In line with this greater disease burden, the event rate and number of AEs were nominally higher in patients in the placebo arm with diabetes than those without diabetes.

Many treatments have shown similar effectiveness in patients with HF regardless of the presence or absence of concomitant diabetes [12]. The present exploratory data suggest that FCM can be added to the list of medications that exhibit benefits in AHF, irrespective of the presence of comorbid diabetes. The lack of interaction between diabetes status and outcomes with FCM vs placebo in AFFIRM-AHF patients is in agreement with previous observations from the FAIR-HF trial, which reported no significant interaction between diabetes status and the beneficial effect of FCM vs placebo on New York Heart Association functional class in patients with chronic HF and iron deficiency [6]. Similarly, CONFIRM-HF reported an improvement in exercise capacity, as measured by 6-minute walk test distance, with FCM vs placebo in patients with chronic HF and iron deficiency, with and without diabetes [27]. Together, these studies suggest that FCM is beneficial in HF patients with iron deficiency, with and without diabetes, irrespective of the type of HF (i.e. acute vs chronic). However, in contrast to the current study, CONFIRM-HF authors also reported a significant interaction between diabetes status and the effect of FCM vs placebo, observing greater improvements in 6-minute walk test distance in patients with diabetes than in those without diabetes [27]. This differential vs the current study may reflect the type of outcome analyzed [27]. Comparison of the results described in the current study with the pending results of the placebo-controlled CLEVER trial—investigating the effect of FCM on HbA_1c_ levels, iron status, and metabolic markers in patients with type 2 diabetes and iron deficiency—will also be of future interest [28].

Despite the higher disease burden and frequency of comorbidities in patients with vs without diabetes, no differences were observed in KCCQ-12 OSS or CSS at baseline. This finding was surprising and in contrast with previous reports of impaired QoL scores in patients with diabetes and HF compared with those without diabetes [8]. This is likely a result of patients in both diabetes and no-diabetes subgroups having experienced a significant life event (an AHF episode) that may have decreased QoL to a similar baseline level. Indeed, following discharge from the hospital, “spontaneous” increases in QoL were seen in the placebo arms of each subgroup over time, but to a lesser extent in patients with diabetes compared with those without diabetes. This may reflect a regression to pre-AHF QoL levels and the negative influence of diabetes on overall QoL. Importantly, the magnitude of the improvement in KCCQ-12 OSS was numerically greater with FCM vs placebo at almost all time points in patients with and without diabetes, with a similar relative effect size at Week 24 and no significant interaction between diabetes status and FCM-related improvements in KCCQ-12 scores. These data suggest that FCM improved overall QoL to a similar magnitude in both patients with and without diabetes. This finding is in agreement with previous observations in the FAIR-HF trial of patients with chronic HF and iron deficiency, which reported no significant interaction between diabetes status and the beneficial effect of FCM on self-reported patient global assessment [6].

Levels of serum ferritin, Hb, and TSAT increased in all treatment arms and subgroups over time, but were increased to a significantly greater magnitude with FCM vs placebo in patients with and without diabetes. This suggests that some spontaneous recovery of iron parameters occurs without iron supplementation following an AHF episode in patients with and without diabetes, but that FCM allows recovery to a greater level in both cases. Changes in iron parameters over time mirrored the improvements in disease-specific QoL, aligning with the well-established relationship between iron deficiency and QoL [6, 20, 27, 29].

Rates of AEs were higher in patients with diabetes compared with those without diabetes, in alignment with the greater disease burden in the former subgroup at baseline. In patients with diabetes, rates of AEs, including treatment-associated AEs and serious AEs, were numerically lower in patients treated with FCM compared with placebo. This may reflect an improvement in overall health following FCM treatment in patients with diabetes.

Several limitations relating to the post hoc*,* exploratory nature of these subgroup analyses should be considered. Firstly, the diabetes and no diabetes subgroups included more modest patient numbers than specified in the overall AFFIRM-AHF power calculations; however, data were available for ≥ 475 patients per subgroup. Secondly, subgroups were based on documented diabetes status (yes/no) plus use of diabetes medication in patient medical records at baseline; further stratification by degree of glycemic control was precluded by a lack of systematic HbA_1c_ data collection. Nevertheless, the varied use of each diabetes medication class in the diabetes subgroup at baseline (including approximately 50% of patients on insulin) suggests that this subgroup represents a spectrum of disease progression and management needs within type 2 diabetes. Thirdly, the potential for heterogeneous glycemic control amongst patients within the diabetes subgroup may have affected results, although the 95% CIs observed for primary, secondary, and QoL outcomes were modest. Future analyses exploring interactions between the extremes of HbA_1c_ (including prediabetes) and the effect of FCM vs placebo on clinical outcomes in AHF patients may be of interest. Longitudinal analyses of HbA_1c_ values over time would also be informative to determine the association between replenishing iron levels and change in HbA_1c_.

## Conclusion

These data suggest that FCM can be used in patients with iron deficiency, with and without diabetes, following an AHF episode to not only reduce clinical events, but also to improve QoL, which is an important outcome from the patient perspective. The high frequency of iron deficiency and diabetes in AHF patients and the associated implications for morbidity and mortality risk highlight the need for both diabetes and iron deficiency screening to enable timely treatment and improved outcomes.

### Supplementary Information

**Additional file 1: Table S1.** First-time initiation of therapy within new diabetes medication class* during the AFFIRM-AHF trial. **Fig S1.** Outcomes in the placebo arms of patients with and without diabetes. **Fig S2.** Outcomes with FCM vs placebo in patients with and without diabetes (COVID-19 sensitivity analysis). **Fig S3.** Iron parameters with FCM vs placebo in patients with and without.

## Data Availability

Full data set available upon reasonable request.

## Abbreviations

AE: Adverse event
AHF: Acute heart failure
CI: Confidence interval
CSS: Clinical summary score
CV: Cardiovascular
eCRF: Electronic clinical report form
FCM: Ferric carboxymaltose
Hb: Hemoglobin
HbA_1c_: Glycated hemoglobin
HF: Heart failure
HR: Hazard ratio
IV: Intravenous
KCCQ-12: 12-Item Kansas City Cardiomyopathy Questionnaire
mITT: Modified intention-to-treat
NA: Not applicable
OSS: Overall summary score
p_interaction_: Interaction p-values
QoL: Quality of life
RR: Rate ratio
SAS: Safety analysis set
SD: Standard deviation
TEAE: Treatment-emergent adverse event
TSAT: Transferrin saturation
