# Supplementary material for: Intravenous ferric carboxymaltose for iron repletion following acute heart failure in patients with and without diabetes: a subgroup analysis of the randomized AFFIRM-AHF trial
Source: Cardiovasc Diabetol. 2023 Aug 17;22:215. doi: 10.1186/s12933-023-01943-z (PMC10436432; doi:10.1186/s12933-023-01943-z)
Supplement: Supplementary file 1 — Additional file 1: Table S1. First-time initiation of therapy within new diabetes medication class* during the AFFIRM-AHF trial. Fig S1. Outcomes in the placebo arms of patients with and without diabetes. Fig S2. Outcomes with FCM vs placebo in patients with and without diabetes (COVID-19 sensitivity analysis). Fig S3. Iron parameters with FCM vs placebo in patients with and without. [file 12933_2023_1943_MOESM1_ESM.pdf]

Additional file appendix

Intravenous ferric carboxymaltose for iron repletion following acute heart failure in patients with and without diabetes:

a subgroup analysis of the randomized AFFIRM-AHF trial

Contents

Supplementary Table 1. First-time initiation of therapy within new diabetes medication class* during the AFFIRM-AHF trial....................2

Supplementary Figure 1. Outcomes in the placebo arms of patients with and without diabetes....................3

Supplementary Figure 2. Outcomes with FCM vs placebo in patients with and without diabetes (COVID-19 sensitivity analysis)....................5

Supplementary Figure 3. Iron parameters with FCM vs placebo in patients with and without diabetes 7

Table S1. First-time initiation of therapy within new diabetes medication class* during the AFFIRM-AHF trial

	**Diabetes (n=475)**		**No diabetes (n=633)**	
	**FCM (n=231)**	**Placebo (n=244)**	**FCM (n=327)**	**Placebo (n=306)**
**Initiation of therapy in new diabetes medication class* during trial, n (%)**				
Insulins and analogues	22 (9.5)	26 (10.7)	6 (1.8)	3 (1.0)
Sodium–glucose cotransporter 2 inhibitor	3 (1.3)	5 (2.0)	0 (0.0)	0 (0.0)
Glucagon-like peptide-1 analogue	2 (0.9)	4 (1.6)	0 (0.0)	0 (0.0)
Dipeptidyl peptidase-4 inhibitor	12 (5.2)	9 (3.7)	0 (0.0)	0 (0.0)
Thiazolidinediones	0 (0.0)	1 (0.4)	0 (0.0)	0 (0.0)

*Includes only patients who were not on a therapy within the specified diabetes medication class at baseline but who initiated a therapy within that medication class during the AFFIRM-AHF trial. Medications with missing or partial dates were imputed assuming the worst case.

FCM, ferric carboxymaltose.

Fig S1. Outcomes in the placebo arms of patients with and without diabetes

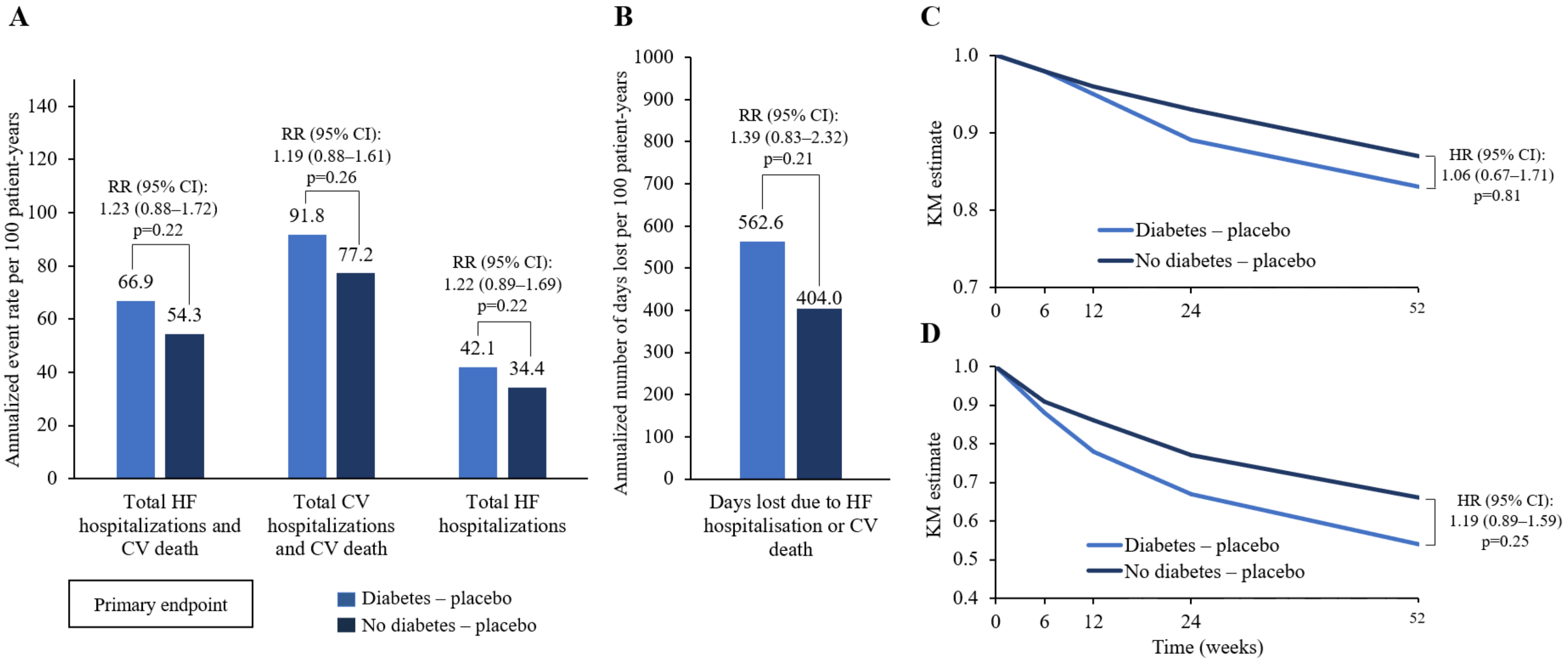

Annualized event rate per 100 patient-years for (A) primary and secondary recurrent event outcomes and (B) days lost due to HF hospitalization or CV death, and Kaplan–Meier estimates for (C) time to CV death and (D) time to first hospitalization or death in the placebo arms of patients with and without diabetes.

Annualized event RR for patients in the placebo group with vs without diabetes analyzed using a negative binomial model. HR for patients in the placebo group with vs without diabetes analyzed using a Cox regression model, with 95% CI provided by the Greenwood's variance estimate. Both models were adjusted for the following baseline covariates: sex, age, HF etiology, HF duration, country (Cox regression model) or region (negative binomial model) and subgroup of diabetic status at baseline. Respective n-numbers for patient with vs without diabetes at baseline assigned to the placebo arm were 244 and 306. CI, confidence interval; CV, cardiovascular; HF, heart failure; HR, hazard ratio; KM, Kaplan–Meier; RR, rate ratio.

Fig S2. Outcomes with FCM vs placebo in patients with and without diabetes (COVID-19 sensitivity analysis)

		Annualized event rate per 100 patient-years[a]		RR or HR[b] (95% CI)	p-value	$p_{interaction}$
**Primary outcome**		FCM	Placebo			
Total HF hospitalizations and CV death	Diabetes	64.5	82.7	RR: 0.78 (0.55–1.12)[a]	0.18	0.82
	No diabetes	49.7	67.4	RR: 0.74 (0.52–1.04)[a]	0.08	
**Secondary outcomes**						
Total CV hospitalizations and CV death	Diabetes	85.6	106.1	RR: 0.81 (0.58–1.12)[a]	0.20	0.76
	No diabetes	68.9	91.7	RR: 0.75 (0.55–1.02)[a]	0.07	
Time to CV death	Diabetes	N/A	N/A	HR: 1.01 (0.64–1.58)[c]	0.97	0.69
	No diabetes	N/A	N/A	HR: 0.88 (0.56–1.41)[c]	0.61	
Total HF hospitalizations	Diabetes	35.0	50.0	RR: 0.70 (0.49–0.99)[a]	0.045	0.91
	No diabetes	28.9	40.1	RR: 0.72 (0.51–1.01)[a]	0.06	
Time to first HF hospitalization or CV death	Diabetes	N/A	N/A	HR: 0.84 (0.63–1.11)[c]	0.22	0.68
	No diabetes	N/A	N/A	HR: 0.77 (0.58–1.03)[c]	0.08	
Days lost due to HF hospitalization or CV death	Diabetes	387.1[d]	650.0[d]	RR: 0.60 (0.34–1.04)[a]	0.07	0.93
	No diabetes	310.4[d]	503.4[d]	RR: 0.62 (0.37–1.02)[a]	0.06	

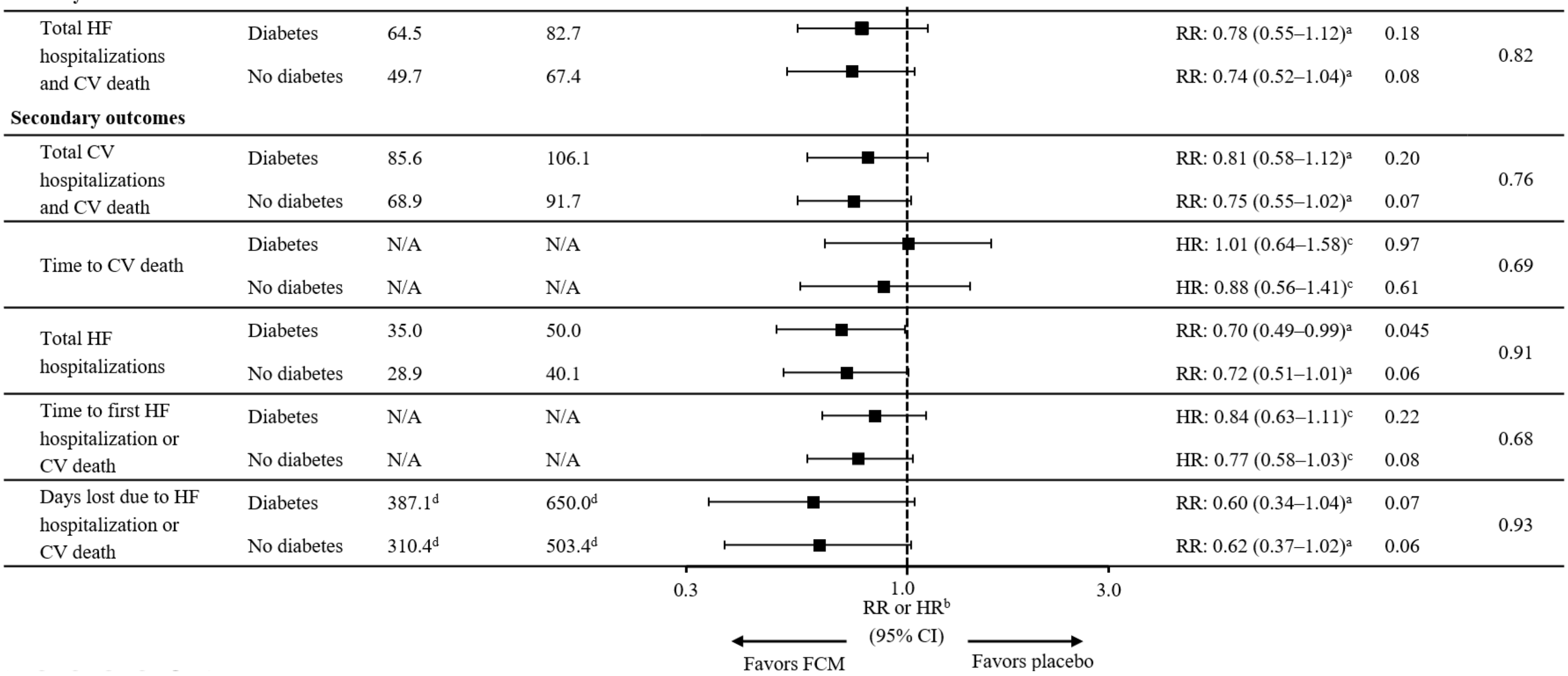

All models adjusted for covariates: sex, age, HF etiology, HF duration, country, and diabetes status at baseline, and diabetes at baseline × treatment. N=1,108 for all patients. Respective n-values in patients with and without diabetes at baseline were 231 and 327 for FCM and 244 and 306 for placebo. [a]Annualized event rate per 100 patient-years and annualized event RR were both analyzed using a negative binomial model. [b]FCM vs placebo. [c]HR for treatment

difference analyzed using Cox regression model. [d]Event refers to day off work. CI, confidence interval; COVID-19, coronavirus disease 2019; CV, cardiovascular; FCM, ferric carboxymaltose; HF, heart failure; HR, hazard ratio; N/A, not applicable; RR, rate ratio.

Fig S3. Iron parameters with FCM vs placebo in patients with and without

A

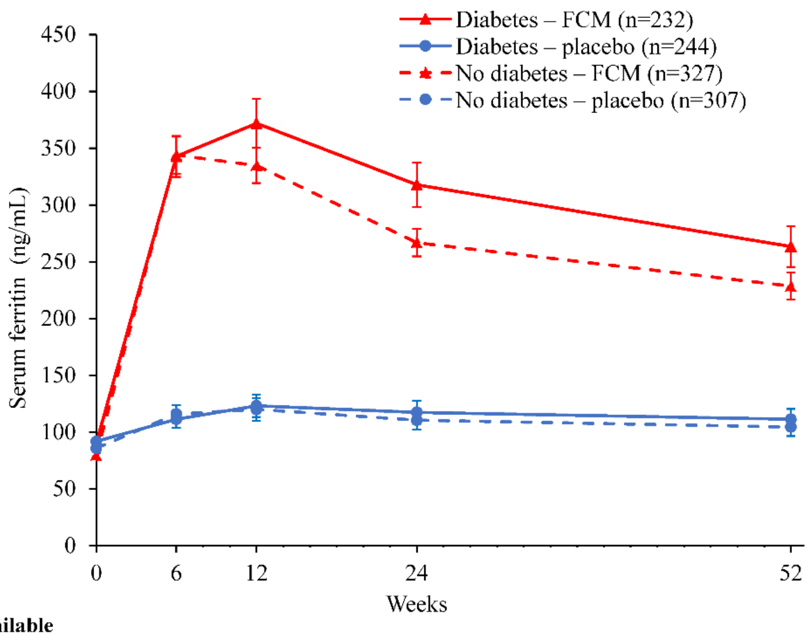

Number available

Diabetes FCM	232	196	174	161	126
Diabetes placebo	244	200	190	184	155
No diabetes FCM	326	280	262	259	213
No diabetes placebo	307	257	248	234	191

B

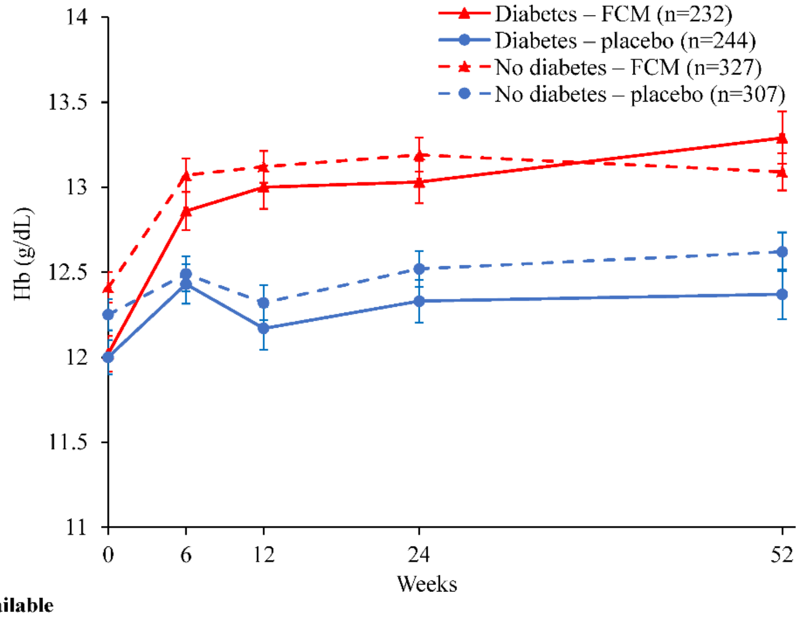

Number available

Diabetes FCM	232	199	174	161	126
Diabetes placebo	244	203	191	184	157
No diabetes FCM	326	281	262	260	213
No diabetes placebo	307	258	248	234	191

C

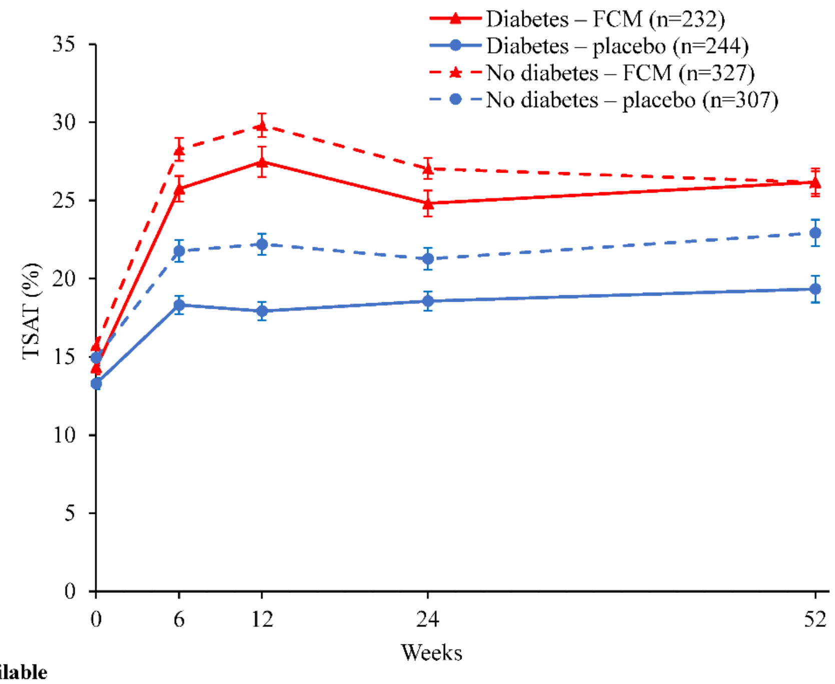

Number available

Diabetes FCM	230	196	169	160	126
Diabetes placebo	241	196	189	183	155
No diabetes FCM	325	280	260	258	212
No diabetes placebo	307	255	246	232	189

Absolute values for serum ferritin (A), hemoglobin (B), and TSAT (C) over time with FCM vs placebo in patients with and without diabetes (safety analysis set population). Error bars are standard error. FCM, ferric carboxymaltose; Hb, hemoglobin; TSAT, transferrin saturation.
